# Isolation and draft genome sequence of *Enterobacter asburiae* strain i6 amenable to genetic manipulation

**DOI:** 10.7150/jgen.91337

**Published:** 2024-01-20

**Authors:** Akinori Kato

**Affiliations:** Department of Advanced Bioscience, Graduate School of Agriculture, Kindai University, 3327-204 Nakamachi, Nara, Nara 631-8505, Japan.

**Keywords:** *Enterobacter asburiae*, gene deletion, λ Red recombinase, plant growth-promoting bacterium (PGPB), cell-cell interactions

## Abstract

*Enterobacter asburiae* is a species of Gram-negative bacteria that is found in soil, water, and sewage. *E. asburiae* is generally considered to be an opportunistic pathogen, but has also been reported as a plant growth-promoting bacterium (PGPB), which may have beneficial effects on plant growth and development. However, genetic analysis of *E. asburiae* has been limited, possibly due to its redundant enzymes that digest exogenous DNA in the cell. Here, an *E. asburiae* strain i6 was isolated from soil in Nara, Japan. This strain was amenable to transformation and the one-step gene inactivation method based on λ Red recombinase. The transformation efficiency of the i6 strain with the 10 kb plasmid DNA pCF430 was at least four orders of magnitude higher than that of the previously sequenced *E. asburiae* strain ATCC 35953, which could not be transformed with the same plasmid DNA. A draft genome sequence of the i6 strain was determined and deposited into the database, allowing several factors that may determine transformation efficiency to be perturbed and tested. Together with the amenability of the i6 strain to genetic manipulation, the information from the i6 genome will facilitate characterization and fine-tuning of the beneficial and detrimental traits of this species.

## Introduction

*Enterobacter asburiae* is a Gram-negative, facultative anaerobic, oxidase-negative, non-motile, and non-pigmented rod-shaped species belonging to the *genus Enterobacter,* which includes species that are difficult to identify with biochemical and phylogenetic tests 1-3. The *Enterobacter cloacae* complex (ECC) is a group of common opportunistic pathogens consisting of *Enterobacter asburiae, Enterobacter cloacae, Enterobacter hormaechei, Enterobacter kobei, Enterobacter ludwigii, Enterobacter mori,* and *Enterobacter nimipressuralis*. In addition, recently identified species, including *Enterobacter roggenkampii, Enterobacter chengduensis*, and *Enterobacter bugandensis*, are clustered with the ECC species 4. While *E. asburiae*, as a part of ECC, is considered to be an opportunistic pathogen, not only *E. asburiae* but also some of *Enterobacter* genus have been reported as plant growth-promoting bacteria (PGPB). Examples include *E. asburiae* PDA 134 from date palm 5, *E. cloacae* from citrus and maize plants 6, 7, and *E. asburiae* from sweet potato 8. *Enterobacter* sp. strain P23 promotes rice growth under salt stress. In addition, *E. mori*, *E. asburiae*, *E. ludwigii*, and *E.* sp. J49 have been shown to promote wheat growth under stress conditions 9. At least some strains of *E. asburiae* reduce the epiphytic fitness of the human enteric pathogens *E. coli* O157:H7 and *Salmonella* on lettuce and *Arabidopsis* by at least 100-fold 9, 10. While* E. asburiae* is a preferable candidate species for genome editing to safely further enhance the ability of PGPB and to study cell-cell interactions, genetic analysis of this important species is limited 11, 12, possibly due to its redundant enzymes that digest exogenous DNA within the cell.

The one-step gene inactivation method based on λ Red recombinase is a powerful and efficient technique used to disrupt specific genes 13 and to construct gene fusions 14 in the bacterial genome. Soon after this method was originally invented in *Escherichia coli* 13, it was widely used to construct deletion mutants and insertion mutants of other Gram-negative species, such as *Salmonella enterica* 15, *Yersinia pestis* 16, *Klebsiella pneumoniae* 17, *Pantoea ananatis* 18. Basically, the technique uses knockout cassettes with short (40-60 bp) homologous arms that can be produced in a single PCR reaction, but some species or strains only accept knockout cassettes with extended arms (200~1,000 bp), which require additional PCR steps to successfully manipulate the gene with λ Red recombinase 19, 20. To date, the one-step gene inactivation method has been unsuccessful with the *E. asburiae* ATCC 35953 (NBRC 109912 ^T^) strain. In addition to genetic factors that reduce the stability of linear DNA fragments, genetic factors that reduce transformation efficiency have generally been thought to hinder genetic manipulations, such as the one-step gene inactivation method. Here, I report a strain of *E. asburiae* i6, isolated from soil in Nara Japan, that is amenable to genetic manipulation.

## Materials and Methods

### Bacterial strains, plasmids, and growth conditions

Bacterial strains and plasmids used in this study are listed in Table 1. Bacteria were grown at 37ºC in LB Broth (Lennox). Ampicillin and spectinomycin were used at 100 µg/ml, kanamycin at 50 µg/ml, chloramphenicol at 25 µg/ml, and tetracycline at 12.5 µg/ml. Primers used in this study are listed in Table 2.

*PCR.* PCRs for 16S and *groEL* were performed using primer sets 63f/1387r 21 and Hsp60-F/Hsp60-R 22 according to their protocols 21, 22. Purified PCR products were analyzed by DNA sequencing.

### Construction of chromosomal gene deletion mutants

Strain AK1602, which has a deletion of the *rcsB* gene and a Cm^R^ cassette, was constructed by the one-step inactivation method 13 using primers A2414 and A2415 with pKD3 as the template.

Strain AK1603, which has a deletion of the *klcA1* gene and a *tetA*, was constructed by the one-step inactivation method 13 using primers A2418 and A2419 with the *phoQ*::Tn*10* chromosomal DNA as the template.

Strain AK1599, which has an insertion of the Km^R^ cassette behind the *wzc* gene, was constructed by the one-step inactivation method 13 using primers A2416 and A2417 with pKD4 as the template.

Strain AK1604, which has a deletion of the *klcA2* gene and a Km^R^ cassette, was constructed by the one-step inactivation method 13 using primers A2420 and A2421 with pKD4 as the template.

Strain AK1605, which has a deletion of the *dgeC* gene and a Km^R^ cassette, was constructed by the one-step inactivation method 13 using primers A2424 and A2425 with pKD4 as the template.

### Plasmid construction

Plasmid RSFRedTER-Sp for λ Bet Exo Gam expression was constructed as a spectinomycin-resistant version of RSFRedTER 18 by the one-step inactivation method 13 using primers A1190 and A1191 with EG16468 chromosomal DNA 23 as the Sp^R^ marker template.

All strains constructed using PCR reactions were analyzed by DNA sequencing to confirm that the PCR-generated DNA regions had the predicted sequences.

### Transformation efficiency analysis

Bacterial cells cultured overnight in LB (Lennox) were added to 10 ml of fresh medium diluted 1:50 in test tubes and shaken at 37°C until the OD_595_ value reached 0.3~0.8. Cells were collected by centrifugation and the supernatant was removed. The cell pellet was washed twice with 1 ml of 10% glycerol, and then the cells were suspended in 400 µl of 10% glycerol. 1-3 µl of plasmid DNA was mixed with 100 µl of competent cells (the cell suspension) and electroporated using a 2 mm cuvette and a Gene Pulser II (Bio-Rad) at 2.5 kV, 200 Ω, and 25 µF. Immediately after the pulse, SOB medium was added to the cuvette and collected in 1.5 ml tubes. Cells were spread on LB (Lennox) plates containing kanamycin (pAK1001 and pACYC177) or tetracycline (pCF430) and incubated at 37°C overnight. Colonies were then counted.

### Genome sequencing, assembly and annotation

Whole genome sequencing was performed using DNBSEQ-G400 (MGI Tech Co., Ltd.). Libraries were prepared using IMGIEasy FS DNA Library Prep Set (MGI Tech Co., Ltd.) and fragments were validated using Fragment Analyzer and dsDNA 915 Reagent Kit (Agilent Technologies). Sequences were de novo assembled using SPAdes assembler version 3.15.5 42. Genome annotation was performed using DFAST.

### Average nucleotide identity analysis

Average nucleotide identity (ANI) analysis 24 was performed using the complete genome sequences of all *Enterobacter* species available in the NCBI database.

## Results and Discussion

### Isolation and genome sequencing of the *Enterobacter asburiae* i6

On July 6, 2016, an *E. asburiae* strain was isolated from the soil near the lawn of the campus of Kindai University Faculty of Agriculture in Nara, Japan, as a strain that somewhat affects the luminescence of a *Salmonella enterica* strain carrying plasmid luciferase reporter under the control of the RcsB-regulated *wza* gene promoter, when cross-streaked. In addition, the colony morphology of this strain is slightly mucoid (or encapsulated) on LB agar plate. 16s rRNA and *groEL* sequencing determined that the strain was approximately *E. asburiae,* and this strain was designated i6. Because the i6 strain may produce a signal molecule that is recognized by *S. enterica* by an unknown mechanism, it would be useful to sequence the genome of strain i6 for future analyses. Therefore, chromosomal DNA was extracted from strain i6 and the genome was sequenced on a next-generation sequencer (DNBSEQ-G400) with X290 coverage. Automated assembly resulted in the formation of 28 contigs, and the longer contigs were dissected by redundant copies of 5s rRNA, 16S rRNA, 23 rRNA, and tRNA. The assembled genome sequences and their annotations were deposited in a database (accession numbers: BTPF01000001~BTPF01000028, DRR495940, PRJDB16388, and SAMD00634955). The sequences of the ATCC 35953, FDAARGOS_892, L1, and RHBSTW-01009 strains, all of which belong to* E. asburiae* (sensu stricto) and not to the four provisional classes (B-E) of *E. asburiae* according to GTDB 25, were the closest to i6 among the complete *E. asburiae* genomes (Table 3). The FDAARGOS_892 strain had the highest ANI value of 98.75%, whereas the L1 strain had the longest average alignment length of 70.08%. Thus, the i6 strain is most likely to be *E. asburiae* (sensu stricto). Alignment of the ends of each contig sequence of i6 to these sequences suggested that the large contigs 1-7, 9, and 10 form an almost complete chromosome of i6 (Table 4), and that contig 8 is a single plasmid DNA itself. Furthermore, the gaps between these contigs were shown to correspond to repetitive 16S rRNA and 23 rRNA, tRNA-Ala, tRNA-Ile, and tRNA-Glu (Table 4).

### Possible genes involved in plant growth promoting traits in the *E. asburiae* i6 genome

Although strain i6 was not originally isolated as PGPB, a list of candidate genes that may contribute to plant growth was compiled from the genome sequence of strain i6 (Table 5). Soil beneficial bacteria can promote plant growth by synthesizing molecules similar to plant hormones 26, 27. Auxin like indole acetic acid (IAA) is quantitatively the most plant hormones secreted by Phytophthora rhizobacteria 28, 29, suggesting that auxin is a signaling molecule in microorganisms 30. Similar to the genome features previously reported for *Enterobacter* sp. J49 30, genes responsible for the synthesis of indole-pyruvate decarboxylase and indole-3-acetaldehyde dehydrogenase were detected in the i6 genome, and no other IAA pathway-related genes were detected. The bacterial volatiles 2,3-butanediol and acetoin are also known to induce plant growth promotion 31. Acetolactate synthase BudB converts pyruvate to acetolactate, which is subsequently converted to acetoin by acetoin decarboxylase BudA. The (S)-acetoin-forming diacetyl reductase BudC catalyzes the conversion of acetoin to 2,3-butanediol, which is reversible. All genes for *butA*, *butB* and *butC* were found, as well as other genes encoding acetolactate synthases (*ilvB*, *ilvN_2*, *ilvG* etc.). Similar to the J49 genome 30, the *gcd* gene encoding GDH synthesis, responsible for the production of gluconic acid, the major organic acid in the phosphate solubilization mechanism most widely used by soil bacteria, was detected in the i6 genome, but the *pqq* gene cluster, required for the biosynthesis of the PQQ cofactor, was not detected. Redundant genes for siderophore-production, iron ABC transporters, and type VI secretion systems which may compete with (plant) pathogens for low iron levels and cell growth, were also found in the i6 genome (data not shown).

### Genes involved in restriction and anti-restriction in the *E. asburiae* i6 genome

**Figure 1:**
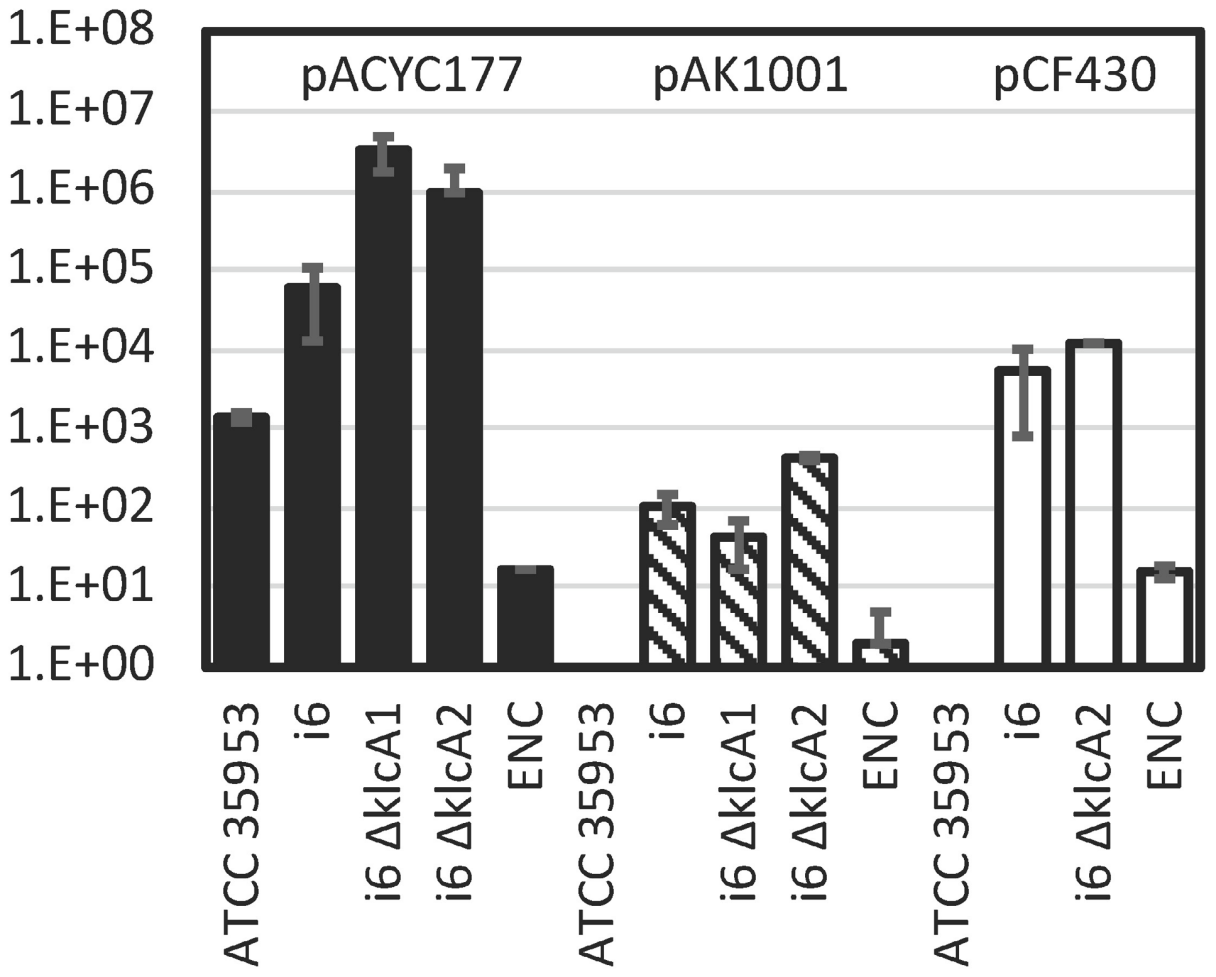
Transformation efficiency of *E. asburiae* strains i6, i6 ∆*klcA1* (AK1603), i6 ∆*klcA2* (AK1604), and ATCC 35953 (NBRC 109912 ^T^) and *E. cloacae* ATCC 1304 (ENC) strain with pACYC177 (dark bar), pAK1001 (gray bar), and pCF430 (white bar). Transformation efficiency (colony count/µg plasmid DNA) was calculated by counting colonies after electroporation, recovery in SOB for 1 h, and overnight growth at 37°C on LB (Lennox) plates containing kanamycin (pACYC177 and pAK1001) or tetracycline (pCF430).

Interestingly, KlcA1 and KlcA2, two homologues of KlcA, which have been reported to function as an anti-type I restriction modification (RM) system in other species 32, were predicted on this genome and plasmid respectively, but only one type IV R enzyme was predicted to be encoded and no type I RM system (Table 6). This is in contrast to other *E. asburiae* strains ATCC 35953, FDAARGOS_892, and RHBSTW-01009, which encode two type I RM systems, one type IV restriction enzyme, and KlcA1 (Table 6). L1 also encodes a type I RM system, a type IV R enzyme, and other restriction systems, but no KlcA homologs. Because it is important for successful genetic manipulation to suppress restriction enzymes and allow bacteria to introduce foreign plasmids, the transformation efficiency of several plasmid DNAs was tested by electroporation using the i6 and ATCC 35953 strains (Fig. 1).

The results showed that the ATCC 35953 strain could not be transformed by the relatively large plasmid DNAs pCF430 (10 kb) 33 and pAK1001 (8.4 kb) 34, whereas i6 formed colonies, showing transformation efficiency at least four orders of magnitude higher than that of ATCC 35953 especially with pCF430 (Fig. 1). Using a relatively small plasmid DNA, pACYC177 (3.9 kb) 35, the ATCC 35953 strain also formed colonies on LB plates containing kanamycin, and the transformation efficiency of i6 was two orders of magnitude higher than that of ATCC 35953 (Fig. 1).

### Application of the one-step gene inactivation method to the i6 strain

Unlike the ATCC 35953 strain, the i6 strain was able to accept foreign DNA with high efficiency (Fig. 1), so I attempted to apply the one-step gene inactivation method 36. Prior to this, RSFRedTER-Sp was constructed by replacing the Cm^R^ marker of the RSFRedTER plasmid 18, which can express λ Red recombinase, with the Sp^R^ marker, so that the Cm^R^ marker could be used in addition to the Km^R^ and Tc^R^ markers in the i6 strain. (Ap-resistant pKD46 was not used because two β-lactamase genes were predicted in the i6 genome.) Gene deletions in *klcA1*, *klcA2,* and *dgeC* were constructed in the i6 strain expressing λ Red recombinase from RSFRedTER-Sp using Tc^R^, Cm^R^, and Km^R^ markers, respectively. Similarly, a deletion in *rcsB* and a Km^R^ insertion behind *wzc* were made in the i6 strain using Cm^R^ and Km^R^ markers, respectively. The recombinants of interest grew with high colony formation rates (Table 7). Colony PCR in the junction region of the recombination site confirmed that the desired recombinants were constructed with high accuracy (Table 7). The ∆*klcA1 and* ∆*klcA2* strains were included in the transformation efficiency analysis because KlcA was originally reported as a restriction enzyme inhibitor in other bacteria, such as *E. coli and Klebsiella pneumoniae* 32*,* and may have contributed to the high transformation efficiency of the plasmid DNA of strain i6*.* However, contrary to expectations, these deletions increased rather than decreased the transformation efficiency of some plasmid DNA (Fig. 1). Yet, this result may have been confounded by the fact that the type I RM system, a potential target of KlcAs, is not encoded in the i6 genome. In conclusion, this report successfully applied the one-step gene inactivation method to the newly identified *E. asburiae* strain i6, whose transformation efficiency was much higher than that of ATCC 35953.

Among the ECC, *E. cloacae* and *E. hormaechei* are the most frequently isolated species in clinical infections, especially in immunocompromised patients and those admitted to intensive care units 37, whereas *E. asburiae* had lower survival rates against serum than *E. cloacae, E. hormaechei*, and *E. ludwigii* isolates 4. In this report, the *E. asburiae* i6 genome unexpectedly exhibited most, if not all, of the genetic features of PGPB previously reported in *Enterobater* sp. J49. This was also true for the genome closest to i6, ATCC 35953, FDAARGOS_892, L1, RHBSTW-01009 (data not shown). Thus, *E. asburiae* is a preferred PGPB candidate in the ECC. Even if the current form of the i6 strain is not optimal in promoting plant growth, it would be advantageous and useful as a starting material for testing and fine-tuning any beneficial and detrimental traits. This is because the i6 strain, with all its potential as a PGPB, can be successfully genetically engineered. First of all, potential risk factors such as genes responsible for antibiotic resistance, biofilm formation, and some of the type VI secretion apparatus/effectors, etc. should be deleted by the one-step gene disruption method or its derivative, scarless genome editing 38. Such a risk negative i6 derivative strain could then be chemically mutagenized to express PGPB factors at optimal levels. Alternatively, or in addition, the ability of strain i6 to be a PGPB can be further enhanced by incorporating plasmid clones harboring other PGPB factors, even from different species, but only as genetically modified organisms. On the other hand, the amenability of this strain must also be useful to identify the relevant genes by deleting candidate genes from this genome that could produce a signal molecule that activates RcsB in *Salmonella* or even within the i6 strains. The recently developed conjugation-mediated versatile site-specific single-copy luciferase fusion system 39, which is broadly applicable to Gram-negative bacteria, should also be helpful in detecting intercellular interactions between the i6 strain and *Salmonella,* etc., and even within the i6 strains.

### Nucleotide Sequence Accession Number

The draft genome sequence of *E. asburiae* i6 has been deposited in the DDBJ/EMBL/GenBank databases under accession numbers BTPF01000001 to BTPF01000028. The raw sequence reads were deposited in DDBJ under BioProject number PRJDB16388 and BioSample number SAMD00634955.

## Figures and Tables

**Table 1:** Bacterial strains and plasmids used in this study.

Strain or plasmid	Description	Reference or source
*E. asburiae*		
109912 ^T^(ATCC 35953)	Wild type	NBRC
i6	Wild type	This work
AK1599	i6 *wzc-*FRT-Km^R^-FRT	This work
AK1602	i6 ∆*rcsB*::FRT-Cm^R^-FRT	This work
AK1603	i6 ∆*klcA1*::Tc^R^	This work
AK1604	i6 ∆*klcA2*::FRT-Km^R^-FRT	This work
AK1605	i6 ∆*dgeC*::FRT-Km^R^	This work
*E. coli*		
DH5α	F^-^ Φ80d*lacZ*ΔM15 Δ(*lacZYA-argF*)U169 *deoR recA*1 *endA*1 *hsdR*17(*r_K_*^-^ *m_K_*^+^) *phoA supE*44 *thi*-1 *gyrA*96 *relA*1	Laboratory stock
JM109λ*pir*	*recA1 endA1 gyrA96 thi1 hsdR17*(*r_K_*^-^ *m_K_*^+^) *e14*^-^ (*mcrA*^-^) *supE44 relA1* Δ(*lac-proAB*)/F' [*traD36*, *proAB*^+^, *lacI^q^*, *lacZ*ΔM15] λ*pir*	Laboratory stock
*Salmonella enterica*		
MS5996	*phoQ*::Tn*10*	40
EG16468	∆P*pmrD-pmrD*::Sp^R^	23
Plasmids		
pKD3	repR6Kγ Ap^R^ FRT Cm^R^ FRT	36
pKD4	repR6Kγ Ap^R^ FRT Km^R^ FRT	36
pCP20	rep_pSC101ts_ Ap^R^ Cm^R^ cl857 λPR flp	41
pCF430	rep_RK2_ *oriT*_RK2_ p_BAD_ *araC* Tc^R^	33
pACYC177	rep_p15A_ Ap^R^ Km^R^	35
RSFRedTER	rep_RSF1010_ *oriV*_RSF1010_ *lacI sacB* Cm^R^ γ β exo	18
RSFRedTER-Sp	rep_RSF1010_ *oriV*_RSF1010_ *lacI sacB* Sp^R^ γ β exo	This work
pAK1001	rep*_oriS_*, Cm^R^, FRT-Km^R^-FRT	34

**Table 2:** Primers used in this study.

Primer name	Sequence
63f	CAGGCCTAACACATGCAAGTC
1387r	GGGCGGWGTGTACAAGGC
Hsp60-F	GGTAGAAGAAGGCGTGGTTGC
Hsp60-R	ATGCATTCGGTGGTGATCATCAG
A1190	CAGCATCCTTGAACAAGGACAATTAACAGTTAACAAATAAGCTGTAATGCAAGTAGCG
A1191	AGGTGGGACCACCCGCGCTACTGCCGCCAGGCAAAGAATCTTTATTTGCCGACTACCTTGA
A2414	CTCCCCTCTGGGGAGAGGGTTAGGGTGAGGGGGATTTTTAGTGTAGGCTGGAGCTGCTTC
A2415	CGGCTATTACGAGTACGAATATAAATCTGACAGCAAATAACATATGAATATCCTCCTTAG
A2416	AAAAGCTTGCTGTAGCAAGGTAGCCTTATACATGAACAATGTGTAGGCTGGAGCTGCTTC
A2417	TCCCCGCGGGAGAGGGACGGGGTGAGACACCCGTCCGGGACATATGAATATCCTCCTTAG
A2418	GCGATGCCGGAACCGAGAATATGTTAACCGTGGAGGATCTGTGTAGGCTGGAGCTGCTTC
A2419	TCTCCTTTAGTCGGGCGGCATCGCCGCCCGTGGTCAGTCACATATGAATATCCTCCTTAG
A2420	CGGGCATTAAACAACAGCACCAGCATAAGGAAATAGTTTACTCTAATGCGCTGTTAATCACT
A2421	GCACCGGGGATAATGCAGCATTCATGTTCGGCATTCCTCACTAAGCACTTGTCTCCTGTT
A2424	AATTATCAAATTCACGCGTTCGGACATCTTCCCTGACGGCGGAATAGGAACTTCAAGATC
A2425	GCTGCTGCATATCTGGTATACCGACGGCTGGACGCCGTCACATATGAATATCCTCCTTAG

**Table 3:** Average nucleotide identity analysis (ANI) data calculated from the nearly complete genome of *Enterobacter asburiae* i6 strain and the whole genome sequence of each *Enterobacter* strains.

Bacterial strains	Total length (bp)	Average aligned length (bp)	Average aligned length (%) respect to *Enterobacter asburiae* i6	ANI value (%) respect to *Enterobacter asburiae* i6
*Enterobacter asburiae* i6	4,623,660	4,623,660	100.00	100.00
*Enterobacter asburiae* **(sensu stricto)** FDAARGOS_892	4,784,820	3,045,120	65.86	98.75
*Enterobacter asburiae* **(sensu stricto)** ATCC 35953	4,804,200	3,122,446	67.53	98.71
*Enterobacter asburiae* **(sensu stricto)** RHBSTW-01009	4,652,220	2,968,019	64.19	98.67
*Enterobacter asburiae* **(sensu stricto)** L1	4,626,720	3,240,164	70.08	98.63
*Enterobacter asburiae* **(B)** MNCRE14	4,491,060	3,000,470	64.89	97.01
*Enterobacter asburiae* **(B)** UBA11899	4,111,620	2,892,021	62.55	96.99
*Enterobacter asburiae* **(B)** 17Nkhm-UP2	4,709,340	3,041,453	65.78	96.95
*Enterobacter asburiae* **(B)** UBA8264	4,433,940	3,091,305	66.86	96.93
*Enterobacter asburiae* **(B)** 1808-013	4,769,520	3,236,906	70.01	96.91
*Enterobacter asburiae* **(D)** R_A5.MM	4,837,860	3,144,464	68.01	95.90
*Enterobacter asburiae* **(C)** TN152	5,042,880	2,899,550	62.71	95.22
*Enterobacter roggenkampii* DSM 16690	4,899,060	3,026,517	65.46	93.03
*Enterobacter chengduensis* WCHECl-C4	5,183,640	2,917,804	63.11	93.21
*Lelliottia nimipressuralis* 51 (*Enterobacter nimipressuralis*)	4,862,340	2,888,082	62.46	92.70
*Enterobacter bugandensis* EB-247	4,717,500	2,825,931	59.90	91.52
*Enterobacter mori* ACYC.E9L	4,807,260	2,814,942	60.88	90.17
*Enterobacter cloacae subsp. cloacae* ATCC 13047 (GCF_000025565.1)	5,596,740	2,783,201	60.19	88.73
*Enterobacter ludwigii* EN-119	4,952,100	2,777,488	56.09	88.54
*Enterobacter hormaechei* ATCC 49162	4,884,780	2,696,351	55.20	87.69
*Enterobacter asburiae* **(E)** INSAq146	4,456,380	2,514,955	54.39	86.68

GTDB 25 categories for *E. asburiae*, sensu stricto or provisional classes (B-E), are indicated in bold.

**Table 4:** Contig sequences and gaps in *E. asburiae* i6 assigned to the complete genome of the closest *E. asburiae* strains.

**Bacterial strain**	***Enterobacter asburiae* (sensu stricto) ATCC 35953**									
i6 contig#	1	2	4	7	5 comp	9 comp	10	6	3 comp	1
Aligned 5'-end position on ATCC 35953 (nt)	0	1691756	2687714	3150068	3315195	3588808	3689839	3776714	3973404	4652519
Aligned 3'-end position on ATCC 35953 (nt)	1691839	2683077	3144943	3310295	3583909	3684694	3771799	3968594	4647866	4713742
Gap to right next contig (nt)	-83	4637	5125	4900	4899	5145	4915	4810	4653	
Gene assigned at gap right next contig		23S rRNA	23S rRNA	23S rRNA	16S rRNA	16S rRNA	16S rRNA	16S rRNA	16S rRNA	
		tRNA-Ala	tRNA-Glu	16S rRNA	tRNA-Glu	tRNA-Ile	tRNA-Ile	tRNA-Glu	tRNA-Glu	
		tRNA-Ile	16S rRNA		23S rRNA	tRNA-Ala	tRNA-Ala	23S rRNA	23S rRNA	
		16S rRNA			5S rRNA	23S rRNA	23S rRNA			
						5S rRNA				
**Bacterial strain**	***Enterobacter asburiae* (sensu stricto) FDAARGOS_892**									
i6 contig#	1 comp	4	7	5 comp	9 comp	10	6	3 comp	2 comp	1 comp
Aligned 5'-end position on FDAARGOS_892 (nt)	0	17644	479998	645123	918757	1019791	1106666	1303359	1981865	2972903
Aligned 3'-end position on FDAARGOS_892 (nt)	12628	474876	640226	913839	1014646	1101753	1298549	1977602	2972986	4717539
Gap to right next contig (nt)	5016	5122	4897	4918	5145	4913	4810	4263	-83	
Gene assigned at gap right next contig	23S rRNA	23S rRNA	23S rRNA	16S rRNA	16S rRNA	16S rRNA	16S rRNA	16S rRNA		
	tRNA-Ala	tRNA-Glu	16S rRNA	tRNA-Glu	tRNA-Ile	tRNA-Ile	tRNA-Glu	tRNA-Glu		
	tRNA-Ile	16S rRNA		23S rRNA	tRNA-Ala	tRNA-Ala	23S rRNA	23S rRNA		
	16S rRNA			5S rRNA	23S rRNA	23S rRNA				
					5S rRNA					
**Bacterial strain**	***Enterobacter asburiae* (sensu stricto) L1**									
i6 contig#	3 comp	2 comp	1 comp	4	7	5 comp	9 comp	10	6	3 comp
Aligned 5'-end position on L1 (nt)	0	482989	1332744	3039704	3527433	3673798	3956299	4060595	4147496	4348070
Aligned 3'-end position on L1 (nt)	478726	1332827	3034691	3522308	3668651	3951379	4055451	4142585	4343261	4561905
Gap to right next contig (nt)	4263	-83	5013	5125	5147	4920	5144	4911	4809	
Gene assigned at gap right next contig	16S rRNA		23S rRNA	23S rRNA	5S rRNA	16S rRNA	16S rRNA	16S rRNA	16S rRNA	
	tRNA-Glu		tRNA-Ala	tRNA-Glu	23S rRNA	tRNA-Glu	tRNA-Ile	tRNA-Ile	tRNA-Glu	
	23S rRNA		tRNA-Ile	16S rRNA	16S rRNA	23S rRNA	tRNA-Ala	tRNA-Ala	23S rRNA	
			16S rRNA			5S rRNA	23S rRNA	23S rRNA		
							5S rRNA			
**Bacterial strain**	***Enterobacter asburiae* (sensu stricto) RHBSTW-01009**									
i6 contig#	5	7 comp	4 comp	1	2	3	6 comp	10 comp	9	5
Aligned 5'-end position on RHBSTW-01009 (nt)	0	252913	396771	889563	2497134	3403492	4148822	4353708	4440814	4541619
Aligned 3'-end position on RHBSTW-01009 (nt)	248012	391646	884543	2495763	3399229	4144010	4348790	4435670	4536700	4586750
Gap to right next contig (nt)	4901	5125	5020	1371	4263	4812	4918	5144	4919	
Gene assigned at gap right next contig	16S rRNA	16S rRNA	16S rRNA	Lrp/AsnC family transcriptional regulator	23S rRNA	23S rRNA	23S rRNA	5S rRNA	5S rRNA	
	23S rRNA	tRNA-Glu	tRNA-Ile	DMT family transporter	tRNA-Glu	tRNA-Glu	tRNA-Ala	23S rRNA	23S rRNA	
		23S rRNA	tRNA-Ala		16S rRNA	16S rRNA	tRNA-Ile	tRNA-Ala	tRNA-Glu	
			23S rRNA				16S rRNA	tRNA-Ile	16S rRNA	
								16S rRNA		

comp: complementary.

**Table 5:** Genes in the genome of *E. asburiae* i6 strain that may contribute to plant growth promotion.

gene	locus_tag	gene product	function
*ipdC*	EAI6_22530	indolepyruvate decarboxylase	phytohormone synthesis
*iaaH*	EAI6_10770	Indole-3-acetyl-aspartic acid hydrolase	indole acetic acid (IAA) synthesis
*budA*	EAI6_03130	acetolactate decarboxylase	acetoin synthesis
*budB*	EAI6_03140	acetolactate synthase AlsS	acetoin synthesis
*budC*	EAI6_03150	(S)-acetoin forming diacetyl reductase	2,3-butanediol synthesis
*-*	EAI6_31530	acetolactate synthase small subunit	acetoin synthesis
*-*	EAI6_31540	acetolactate synthase 3 large subunit	acetoin synthesis
*ilvB*	EAI6_35410	acetolactate synthase large subunit	acetoin synthesis
*ilvN_2*	EAI6_35420	acetolactate synthase small subunit	acetoin synthesis
*ilvG*	EAI6_42550	acetolactate synthase 2 catalytic subunit	acetoin synthesis
*ilvM*	EAI6_42560	acetolactate synthase 2 small subunit	acetoin synthesis
*gcd*	EAI6_31050	quinoprotein glucose dehydrogenase	gluconic acid synthesis

**Table 6:** Highly identical (>90%) ortholog list of restriction enzymes and antirestriction proteins among *E. asburiae* strains.

	*Enterobacter asburiae* (sensu stricto) strain				
	i6	L1	ATCC 35953	FDAARGOS_892	RHBSTW-01009
gene product	locus_tag				
antirestriction protein (KlcA1)	EAI6_00720	ND	ACJ69_22495	I6G49_09840	HV349_19355
antirestriction protein (KlcA2)	EAI6_40610	ND	ND	ND	ND
type IV restriction enzyme	EAI6_32920	ND	ND	ND	ND
type IV restriction enzyme	ND	ND	ACJ69_09575	I6G49_13575	ND
PD-(D/E)XK nuclease superfamily protein	ND	ND	ACJ69_09580	I6G49_13570	ND
**type I restriction enzyme, R subunit**	ND	ND	**ACJ69_09595**	**I6G49_13555**	**HV349_13365**
type I restriction enzyme, S subunit	ND	ND	ACJ69_09610	I6G49_13540	HV349_13375*
type I restriction enzyme M protein	ND	ND	ACJ69_09615	I6G49_13535	HV349_13390
type II restriction enzyme M protein	ND	ND	ACJ69_21260	I6G49_08600	HV349_10105/HV349_12830
type I restriction enzyme M protein	ND	ND	ACJ69_22055	I6G49_09395	ND
type I restriction enzyme, S subunit	ND	ND	ACJ69_22060	I6G49_09400	ND
**type I restriction enzyme, R subunit**	ND	ND	**ACJ69_22070**	**I6G49_09410**	ND
**type I restriction enzyme, R subunit**	ND	ND	ND	ND	**HV349_19450**
type I restriction enzyme, S subunit	ND	ND	ND	ND	HV349_13375
type I restriction enzyme M protein	ND	ND	ND	ND	HV349_13380
5-methylcytosine-specific restriction enzyme B	ND	DI57_15815	ND	ND	ND
5-methylcytosine-specific restriction enzyme subunit McrC	ND	DI57_15820	ND	ND	ND
putative restriction endonuclease, HNH endonuclease	ND	DI57_15875	ND	ND	ND
restriction methylase	ND	DI57_15880	ND	ND	ND
Type IV restriction system protein	ND	DI57_15975	ND	ND	ND
**type I restriction enzyme, R subunit**	ND	**DI57_15980**	ND	ND	ND
type I restriction enzyme, S subunit	ND	DI57_15985	ND	ND	ND
type I restriction enzyme M protein	ND	DI57_15990	ND	ND	ND

ND: not detected. *Identity was less than 90%. Type I restriction enzyme, R subunit was shown in bold.

**Table 7:** Recombination efficiency and accuracy for deletion and insertion of targeted genes in the i6 strain

Target gene	Chromosome or plasmid	Deletion or insertion	Marker	Number of recombinants	Targeting success rate (%)
*klcA1*	chromosome	deletion	Tc^R^	289	100 (8/8)
*klcA2*	plasmid	deletion	Cm^R^	143	100 (8/8)
*dgeC*	chromosome	deletion	Km^R^	48	100 (8/8)
*wzc*	chromosome	insertion	Km^R^	6	100 (6/6)

## References

[1] Paauw A, Caspers MP, Schuren FH, Leverstein-van Hall MA, Deletoile A, Montijn RC (2008). Genomic diversity within the *Enterobacter cloacae* complex. PLoS One.

[2] Humann JL, Wildung M, Pouchnik D, Bates AA, Drew JC, Zipperer UN (2014). Complete genome of the switchgrass endophyte *Enterobacter clocace* P101. Stand Genomic Sci.

[3] Brady C, Cleenwerck I, Venter S, Coutinho T, De Vos P (2013). Taxonomic evaluation of the genus *Enterobacter* based on multilocus sequence analysis (MLSA): proposal to reclassify *E nimipressuralis* and *E amnigenus* into *Lelliottia* gen nov as *Lelliottia nimipressuralis* comb nov and *Lelliottia amnigena* comb nov, respectively, *E gergoviae* and *E pyrinus* into *Pluralibacter* gen nov as *Pluralibacter gergoviae* comb nov and *Pluralibacter pyrinus* comb nov, respectively, *E cowanii, E radicincitans, E oryzae* and *E arachidis* into *Kosakonia* gen nov as *Kosakonia cowanii* comb nov, *Kosakonia radicincitans* comb nov, *Kosakonia oryzae* comb nov and *Kosakonia arachidis* comb nov, respectively, and *E turicensis, E helveticus* and *E pulveris* into *Cronobacter* as *Cronobacter zurichensis* nom nov, *Cronobacter helveticus* comb nov and *Cronobacter pulveris* comb nov, respectively, and emended description of the genera *Enterobacter* and *Cronobacter*. Syst Appl Microbiol.

[4] Ganbold M, Seo J, Wi YM, Kwon KT, Ko KS (2023). Species identification, antibiotic resistance, and virulence in *Enterobacter cloacae* complex clinical isolates from South Korea. Front Microbiol.

[6] Araujo WL, Marcon J, Maccheroni W Jr, Van Elsas JD, Van Vuurde JW, Azevedo JL (2002). Diversity of endophytic bacterial populations and their interaction with *Xylella fastidiosa* in citrus plants. Appl Environ Microbiol.

[7] Hinton DM, Bacon CW (1995). *Enterobacter cloacae* is an endophytic symbiont of corn. Mycopathologia.

[8] Asis CA Jr, Adachi K (2004). Isolation of endophytic diazotroph *Pantoea agglomerans* and nondiazotroph *Enterobacter asburiae* from sweetpotato stem in Japan. Lett Appl Microbiol.

[9] Zhang G, Sun Y, Sheng H, Li H, Liu X (2018). Effects of the inoculations using bacteria producing ACC deaminase on ethylene metabolism and growth of wheat grown under different soil water contents. Plant Physiol Biochem.

[10] Li G, Hu Z, Zeng P, Zhu B, Wu L (2015). Whole genome sequence of *Enterobacter ludwigii* type strain EN-119T, isolated from clinical specimens. FEMS Microbiol Lett.

[11] Bi C, Zhang X, Ingram LO, Preston JF (2009). Genetic engineering of *Enterobacter asburiae* strain JDR-1 for efficient production of ethanol from hemicellulose hydrolysates. Appl Environ Microbiol.

[12] Edoamodu CE, Nwodo UU (2022). Marine sediment derived bacteria *Enterobacter asburiae* ES1 and *Enterobacter* sp. Kamsi produce laccase with high dephenolisation potentials. Prep Biochem Biotechnol.

[13] Datsenko KA, Wanner BL (2000). One-step inactivation of chromosomal genes in *Escherichia coli* K-12 using PCR products. Proc Natl Acad Sci U S A.

[14] Ellermeier CD, Janakiraman A, Slauch JM (2002). Construction of targeted single copy lac fusions using lambda Red and FLP-mediated site-specific recombination in bacteria. Gene.

[15] Uzzau S, Figueroa-Bossi N, Rubino S, Bossi L (2001). Epitope tagging of chromosomal genes in *Salmonella*. Proc Natl Acad Sci U S A.

[16] Winfield MD, Latifi T, Groisman EA (2005). Transcriptional regulation of the 4-amino-4-deoxy-L-arabinose biosynthetic genes in *Yersinia pestis*. J Biol Chem.

[17] Mitrophanov AY, Jewett MW, Hadley TJ, Groisman EA (2008). Evolution and dynamics of regulatory architectures controlling polymyxin B resistance in enteric bacteria. PLoS Genet.

[18] Katashkina JI, Hara Y, Golubeva LI, Andreeva IG, Kuvaeva TM, Mashko SV (2009). Use of the lambda Red-recombineering method for genetic engineering of *Pantoea ananatis*. BMC Mol Biol.

[19] Derbise A, Lesic B, Dacheux D, Ghigo JM, Carniel E (2003). A rapid and simple method for inactivating chromosomal genes in *Yersinia*. FEMS Immunol Med Microbiol.

[20] Lesic B, Rahme LG (2008). Use of the lambda Red recombinase system to rapidly generate mutants in *Pseudomonas aeruginosa*. BMC Mol Biol.

[21] Marchesi JR, Sato T, Weightman AJ, Martin TA, Fry JC, Hiom SJ (1998). Design and evaluation of useful bacterium-specific PCR primers that amplify genes coding for bacterial 16S rRNA. Appl Environ Microbiol.

[22] Hoffmann H, Roggenkamp A (2003). Population genetics of the nomenspecies *Enterobacter cloacae*. Appl Environ Microbiol.

[23] Kato A, Latifi T, Groisman EA (2003). Closing the loop: the PmrA/PmrB two-component system negatively controls expression of its posttranscriptional activator PmrD. Proc Natl Acad Sci U S A.

[24] Richter M, Rossello-Mora R (2009). Shifting the genomic gold standard for the prokaryotic species definition. Proc Natl Acad Sci U S A.

[25] Parks DH, Chuvochina M, Rinke C, Mussig AJ, Chaumeil PA, Hugenholtz P (2022). GTDB: an ongoing census of bacterial and archaeal diversity through a phylogenetically consistent, rank normalized and complete genome-based taxonomy. Nucleic Acids Res.

[26] Glick BR (2012). Plant growth-promoting bacteria: mechanisms and applications. Scientifica (Cairo).

[27] Coulson TJ, Patten CL (2015). Complete Genome Sequence of *Enterobacter cloacae* UW5, a Rhizobacterium Capable of High Levels of Indole-3-Acetic Acid Production. Genome Announc.

[28] Kim B, Park AR, Song CW, Song H, Kim JC (2022). Biological Control Efficacy and Action Mechanism of *Klebsiella pneumoniae* JCK-2201 Producing Meso-2,3-Butanediol Against Tomato Bacterial Wilt. Front Microbiol.

[29] Chandarana KA, Amaresan N (2023). Predation pressure regulates plant growth promoting (PGP) attributes of bacterial species. J Appl Microbiol.

[30] Luduena LM, Anzuay MS, Angelini JG, McIntosh M, Becker A, Rupp O (2019). Genome sequence of the endophytic strain *Enterobacter* sp. J49, a potential biofertilizer for peanut and maize. Genomics.

[31] Ryu CM, Farag MA, Hu CH, Reddy MS, Wei HX, Pare PW, Kloepper JW (2003). Bacterial volatiles promote growth in *Arabidopsis*. Proc Natl Acad Sci U S A.

[32] Serfiotis-Mitsa D, Herbert AP, Roberts GA, Soares DC, White JH, Blakely GW (2010). The structure of the KlcA and ArdB proteins reveals a novel fold and antirestriction activity against Type I DNA restriction systems *in vivo* but not *in vitro*. Nucleic Acids Res.

[33] Newman JR, Fuqua C (1999). Broad-host-range expression vectors that carry the L-arabinose-inducible *Escherichia coli araBAD* promoter and the *araC* regulator. Gene.

[34] Kato A (2016). *In vivo* cloning of large chromosomal segments into a BAC derivative by generalized transduction and recombineering in *Salmonella enterica*. J Gen Appl Microbiol.

[35] Schottel JL, Bibb MJ, Cohen SN (1981). Cloning and expression in streptomyces lividans of antibiotic resistance genes derived from *Escherichia coli*. J Bacteriol.

[36] Datsenko KA, Wanner BL (2000). One-step inactivation of chromosomal genes in *Escherichia coli* K-12 using PCR products. Proc Natl Acad Sci USA.

[38] Fels U, Gevaert K, Van Damme P (2020). Bacterial Genetic Engineering by Means of Recombineering for Reverse Genetics. Front Microbiol.

[39] Kato A (2023). Development of conjugation-mediated versatile site-specific single-copy luciferase fusion system. J Gen Appl Microbiol.

[40] Fields PI, Groisman EA, Heffron F (1989). A *Salmonella* locus that controls resistance to microbicidal proteins from phagocytic cells. Science.

[41] Cherepanov PP, Wackernagel W (1995). Gene disruption in *Escherichia coli*: Tc^R^ and Km^R^ cassettes with the option of Flp-catalyzed excision of the antibiotic-resistance determinant. Gene.

[42] Prjibelski A (2020). Using SPAdes De Novo Assembler. Curr Protoc Bioinformatics.

